# Inhibitory effect of Zhujing Pill on myopia progression: Mechanistic insights based on metabonomics and network pharmacology

**DOI:** 10.1371/journal.pone.0312379

**Published:** 2024-12-03

**Authors:** Yongle Du, Mengran Pang, Haoyu Chen, Xiangkun Zhou, Ruyue Geng, Yanan Zhang, Linqi Yang, Jiawen Li, Yufeng Han, Jinpeng Liu, Ruixue Zhang, Hongsheng Bi, Dadong Guo

**Affiliations:** 1 Shandong University of Traditional Chinese Medicine, Jinan, China; 2 Affiliated Eye Hospital of Shandong University of Traditional Chinese Medicine, Jinan, China; 3 Shandong Provincial Key Laboratory of Integrated Traditional Chinese and Western Medicine for Prevention and Therapy of Ocular Diseases, Jinan, China; 4 Shandong Academy of Eye Disease Prevention and Therapy, Jinan, China; 5 Medical College of Optometry and Ophthalmology, Shandong University of Traditional Chinese Medicine, Jinan, China; The Ohio State University, UNITED STATES OF AMERICA

## Abstract

**Objectives:**

This study endeavored to uncover the mechanisms by which Zhujing pill (ZJP) slows myopia progression.

**Methods:**

We employed biometric analyses to track diopter and axial length changes in guinea pigs with negative lens-induced myopia (LIM). Through integrating metabonomics and network pharmacology, we aimed to predict the anti-myopic targets and active ingredients of ZJP. Subsequent analysis, including real-time fluorescent quantitative PCR (qPCR) and Western blotting (WB), assessed the expression levels of CHRNA7, LPCAT1, and NOS2 in retinal tissues.

**Key findings:**

Our findings demonstrate that ZJP significantly mitigates diopter increase and axial elongation in LIM guinea pigs. Metabonomic analysis revealed significant changes in 13 serum metabolites, with ZJP reversing the expression of 5 key metabolites. By integrating metabonomics with network pharmacology, we identified core targets of ZJP against myopia and constructed a compound-gene-disease-metabolite network. The expressions of LPCAT1 and CHRNA7 were found to decrease in the LIM group but increase with ZJP treatment, whereas NOS2 expression showed the opposite pattern.

**Conclusions:**

This investigation provides the first evidence of ZJP’s multifaceted effectiveness in managing myopia, highlighting its impact on multiple components, targets, and pathways, including the novel involvement of LPCAT1 and CHRNA7 in myopia pathogenesis.

## Introduction

Myopia stands as a principal contributor to blindness worldwide, marking its emergence as a significant public health challenge. This condition, characterized by its capacity to precipitate a spectrum of visual impairments including choroidal neovascularization and myopic macular degeneration, underscores the urgency of addressing myopia. Forecasts suggest an alarming trend, with projections indicating that by the year 2050, myopia will afflict approximately half of the global population [1]. The etiology of myopia is multifaceted, stemming from an intricate interplay between genetic predispositions and environmental factors [2]. Consequently, understanding the pathophysiological mechanisms underlying myopia and elucidating the pharmacological interventions capable of its mitigation are paramount for its prevention and management.

The elucidation of metabolic biomarkers and signaling pathways has been a pivotal aspect of myopia research. Recent metabonomic analyses, encompassing studies on serum from myopia control, aqueous humor from individuals with high myopia and cataracts, as well as serum from subjects with high myopia, have delineated profound metabolic alterations accompanying myopia’s progression [3]. Despite these advances, the identification of upstream regulatory targets within these metabolic pathways remains an unresolved challenge.

Zhu-Jing-Pill (ZJP), a traditional Chinese medicine formula widely recognized in ophthalmologic applications, comprises an intricate blend of Broussoneria papyrifera, Lycium barbarum, Schisandra chinensis, Cuscuta chinensis, Cistanche deserticola, Zanthoxylum bungeanum, Panax ginseng, Rehmannia glutinosa, and frankincense. Notably, ZJP has been documented to effectively address diabetic retinopathy [4]. High-performance liquid chromatography analyses have successfully identified hyperin, geniposide, and mulberry glycoside within the ZJP composition [5]. Further, the density of macular pigment in individuals with myopia is negatively correlated with intraocular pressure and axial length, indicating that supplementation with Chinese wolfberry could mitigate macular pigment depletion in patients with high myopia [6]. Additionally, in diabetic mouse models, ginseng has been shown to significantly counteract the diabetes-induced upregulation of extracellular matrix proteins and vasoactive factors [7]. This research pioneers in elucidating ZJP’s potential mechanisms in impeding myopia progression.

The development of lens-induced myopia (LIM), which simulates human myopia through the application of a negative lens over the eye, resulting in the elongation of the ocular axis, serves as a model for this condition [8]. Our study aims to delineate the primary targets and conjectured active components of ZJP in the suppression of myopia, utilizing the LIM guinea pig model, integrated with metabonomic and network pharmacological strategies. Validation of these findings was further conducted through real-time quantitative PCR (qPCR) and western blotting (WB), providing a comprehensive understanding of ZJP’s role in myopia management.

## Materials and methods

### ZJP preparation

The ZJP formulation, procured from the Jianlian Traditional Chinese Medicine Pharmacy, is composed of a meticulously selected array of ingredients: Cuscuta chinensis Lam., Schisandra chinensis (Turcz.) Baill., Cistanche deserticola Ma, Zanthoxylum bungeanum Maxim, Fructus Broussonetiae, Panax ginseng C. A. Meyer, Lycium barbarum, Rehmannia glutinosa (Gaertn.) DC., and Frankincense, in the ratios of 10:10:5:10:10:3:10:10:5, respectively. For the extraction process, the blend was immersed in distilled water at tenfold its volume for two hours, followed by a heating and refluxing phase lasting 1.5 hours. The extract was then concentrated using a rotary evaporator set at 50°C. The final ZJP product was formulated into a liquid solution, with a concentration of 1.5 g/ml of the crude drug components, and was preserved under refrigeration at 4°C to ensure stability and efficacy.

### Ethics statement

Committee at Shandong University of Traditional Chinese Medicine approved this work, and all procedures adhere to the ARVO Statement on the Use of Animals in Ophthalmology and Vision Research. The study followed the Guidelines for the Care and Use of Laboratory Animals by the National Institutes of Health. The ethical approval number is AWE-2022-055 (approval date: 5th May, 2022). Animals’ malaise and discomfort were minimized through effort.

### Animals

In this study, healthy male colored guinea pigs, aged 4 weeks, were sourced from Danyang Changyi Experimental Animal Breeding Co., Ltd., located in Jiangsu, China. Prior to enrollment in the study, guinea pigs presenting with any ocular pathologies were rigorously excluded. The environmental conditions for the guinea pigs were carefully controlled, involving a cyclical light-dark regimen consisting of 12 hours of darkness followed by 12 hours of exposure to natural light, with an average illuminance of 450 lux. The ambient noise level was maintained below 55 decibels to minimize stress, and the temperature was kept constant at 24°C ± 2°C to ensure a stable and comfortable habitat for the subjects throughout the duration of the research.

### ZJP administration and Establishment of the LIM guinea pig model

Following a week-long period of acclimatization to their new environment, the guinea pigs were systematically allocated into four distinct groups: the normal control (NC) group, the lens-induced myopia (LIM) group, the ZJP group, and the normal saline (NS) group. The NC group received daily oral administration of physiological saline at a dosage of 5ml/kg, without any treatment applied to either eye. For the induction of myopia in the LIM group, guinea pigs were not subjected to intragastric administration; instead, a -6.00D spherical resin lens was fitted over the right eye for a duration of four weeks. Similarly, the NS group guinea pigs were administered physiological saline (5ml/kg) orally, with a -6.00D spherical resin lens applied to the right eye. In contrast, the ZJP group received daily oral gavage of the ZJP formulation (5ml/kg), with the right eye also being covered with a -6.00D spherical resin lens. To ensure the consistency and effectiveness of the myopia induction process, any lenses that were dislodged were promptly repositioned to maintain the intended experimental conditions for LIM. Following the experiment, all animals were euthanized via anesthesia overdose (intraperitoneal injection of 150 mg/kg of nembutal). The right eyes of the guinea pigs were removed immediately after execution to collect retinal tissues.

### Refractive and A-scan measurements

At the 0 and 4 weeks following the experiment’s commencement, the refractive parameters of the guinea pigs in the four groups were measured, starting with the removal of their lenses. To facilitate full pupil dilation, sodium cyclopentate hydrochloride eye drops from Geneva Alcon, Switzerland, were applied into the conjunctival sac at fifteen-minute intervals four times. Refractive examinations were conducted in a darkened environment 45 minutes after the last application, ensuring that each eye was measured at least six times for accuracy. For the determination of ocular axial length (AL), A-type ophthalmic ultrasonography, specifically the Cinescan by Quantel Medical, Cournon-d’Auvergne, France, was utilized. Hydroxybuprocaine hydrochloride from Osaka, Japan, served as the topical anesthetic before the axial length measurements were taken. By performing ten measurements of axial length and calculating the average value, precise and consistent data regarding the ocular dimensions of the guinea pigs were obtained.

### Serum sample collection

Following four weeks of myopia inducing, ten guinea pigs were assigned at random from each group and euthanized by intraperitoneal injection of 5% pentobarbital. Blood samples were obtained from the orbit of guinea pigs after ocular enucleation. Anticoagulant-free blood was collected in a vacuum vessel. The serum samples were acquired through a 10-min centrifugation at 3000rpm (NEST Biotech., Wuxi, China) and subsequently stored at -80°C.

### LC/MS-based Serum metabolomics

#### Preparation of Serum samples

The serum samples were defrosted and swirled for 30 seconds (NEST Biotech, Wuxi, China). To prepare tissue homogenate, add cold PBS, and mix for 10 minutes at 4°C. To extract metabolites, 200 μL materials were mixed with MeOH and ACN (1:1, v/v). The samples were then swirled for 30 seconds and sonicated for 10 minutes. The precipitated sample was recreated in 40 μL/mg.pro of ACN:H2O (1:1, v/v), swirled for 30 seconds, sonicated for 10 minutes, followed by centrifugation for 15 min at 20,000 rpm and 4°C. The supernatants were moved to HPLC vials and kept at -80°C before LC/MS analysis.

#### LC-MS/MS

Using mobile phase A (water combined with 25 mM ammonium hydroxide and 25 mM ammonium acetate) and mobile phase B (ACN), the samples were separated on an amide column. There was a 4 μL sample size and a 0.4 ml/min current velocity. Both positive and negative ion patterns of the Q-Exactive MS/MS were used for MS analysis.

### Network pharmacology

To elucidate the mechanisms through which ZJP influences the progression of myopia, we employed network pharmacology approaches to identify the targets of ZJP involved in myopia modulation. Utilizing the Traditional Chinese Medicine Systems Pharmacology Database and Analysis Platform (TCMSP) (**https://old.tcmspe.com/tcmsp.php**), we retrieved all chemicals constituent of ZJP. Active components were selected based on criteria of drug-likeness (DL) ≥ 0.18 and oral bioavailability (OB) ≥ 30%. To identify myopia-related targets, we conducted keyword searches for "myopia" within the GeneCards human gene database (GeneCards, **www.genecards.org/**) and the Online Mendelian Inheritance in Man (OMIM, **https://omim.org/**) gene map. Intersection genes between ZJP targets and myopia-associated targets were then identified, analyzed through Kyoto Encyclopedia of Genes and Genomes (KEGG) and Gene Ontology (GO), and integrated into a Protein-Protein Interaction (PPI) network. For pathway and gene ontology (GO) enrichment analysis of the potential targets, we utilized Metascape 3.5 (metascape.org/gp/index.html). Visualization tools including the GO circle diagram, triple bar diagram, and bubble diagram were generated via **https://www.bioinformatics.com.cn**, as of July 10, 2023. The construction of the PPI network was facilitated through Cytoscape 3.9.0-BETA1 and STRING 11.5 (**https://string-db.org/**), with CytoHubba plugin in Cytoscape employed to identify the hub gene. KEGG pathway analysis criteria were set for an enrichment factor >1.5 and a p-value <0.01, ensuring the identification of significant pathways and gene interactions pertinent to the mitigation of myopia progression via ZJP.

### Real-time fluorescent quantitative PCR

Four-week-old NC, LIM, and ZJP guinea pigs had their retinas removed and preserved in sampling tubes (NEST Biotech, Wuxi, China) using liquid nitrogen. Next, using the modified tissue/cell RNA extraction kit (SparkJade Science Co., Ltd., Jinan, China), the same amount of retinal tissue was extracted. Reverse-transcribed cDNA from target genes inducible nitric oxide synthase (NOS2), nicotinic choline receptor α7 (CHRNA7), and lysophosphatidylcholinyltransferase 1 (LPCAT1) was performed by HiScript II Q RT SuperMix (+ gDNAwiper) for qPCR (Vazyme Biotech Co., Ltd., Nanjing, China). Premier 5.0 software was used to develop the primers (Table 1) before Shanghai Sangon Biotechnology Company (Shanghai, China) produced them. Using the LightCycler ®480 II sequence detection equipment (Roche Applied Science, IN, USA), qPCR was conducted using the cDNA of the aforementioned target genes.

**Table 1:** Primer sequences for target genes.

Gene names	Forward primer (5′-3′)	Reverse primer (5′-3′)
CHRNA7	CGGAGCGAGAAGTTCTACGAGT	TGAACACCGTGAGGGACAGG
LPCAT1	GGCTCCTATTTGCCGCTTTC	CAGTGCCTGTCGTCCCTTCA
NOS2	GAAACTCGGAGACCCAAAAGACG	GGTTGAAAGCACAACTGAACAAGG
GAPDH	CTGACCTGCGCCTGGAGAAACC	ATGCCAGCCCCAGCGTCAAAAGT

### Western blotting

After 4 weeks of myopic induction, 3 guinea pigs in each group were randomly chosen to separate the retinal tissue. Total retinal proteins were extracted with RIPA buffer containing PMSF. Use a grinder for grinding. Then the supernatant was collected by 5200rpm centrifugation and 2 min. SDS-PASE color gel extremely rapid preparation kit (SparkJade Science Co., Ltd., Jinan, China), the total protein was subjected to 10% SDS-PAGE. The antibodies were as follows: CHRNA7 (dilution 1:1000; ABclonal Biotechnology), NOS2 (dilution 1:800; ABclonal Biotechnology), and LPCAT1 (dilution 1:1000; Bioss, Beijing, China). A fusion-fx7 imaging system (Vilber Lourmat, Marne-la-Wall é e, France) was used to visualize the combination. Ultimately, DAB (Sigma-Aldrich, St. Louis, MO, USA) and fusion CAPT software (Vilber Lourmat, Marne-la- Wall é e, France) were used to assess the protein expression.

### Statistical analysis

GraphPad Prism 9.0.0 (GraphPad Software, San Diego, CA, USA) was used to conduct statistical analysis. Diopter and axis length were examined using a two-way ANOVA, whereas WB, qPCR, and the normalized signal strength of potential biomarkers were analyzed using a one-way ANOVA with Tukey’s post-hoc test. The experiment was repeated at least three times, and the results were expressed as mean ± SD. P < 0.05 was considered statistically significant.

## Results

### ZJP slows the progression of experimental myopia

**Fig 1:**
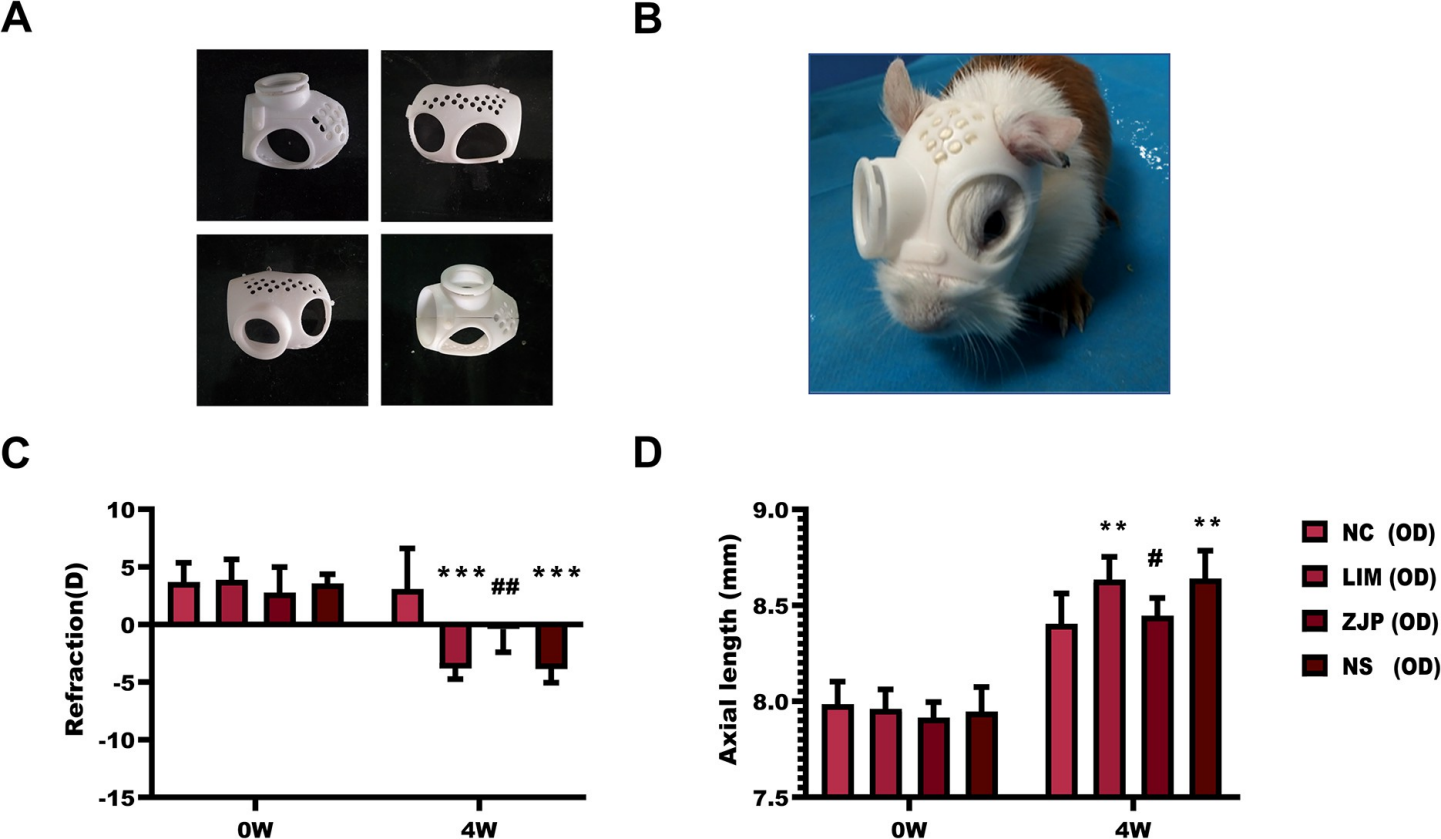
Changes in refraction and axial length of the animals. A, B: Establishment of the LIM guinea pig model. C, D: Measurement of refraction and axial length of the animals. **P < 0.01 and ***P < 0.001 vs. the NC group, ^#^P < 0.05 and ^##^P < 0.01 vs. the LIM group.

To evaluate the anti-myopia effect of ZJP, we detected the diopter and axial length in NC, LIM, ZJP, and NS groups. The diopter and axial length of the three groups did not differ significantly before the induction of myopia (Fig 1, P>0.05). After four weeks of myopia induction, both the LIM and NS groups showed a significant rise in ocular AL (Fig 1D, P<0.01) and a large refractive error when comparing to the NC group (Fig 1C, P<0.001). On the other hand, the ZJP group showed a significant reduction in axial length (Fig 1D, P<0.05) and diopter (Fig 1C, P<0.01) as compared to the LIM group.

### ZJP may slow the progression of myopia by modulating critical metabolites

**Fig 2:**
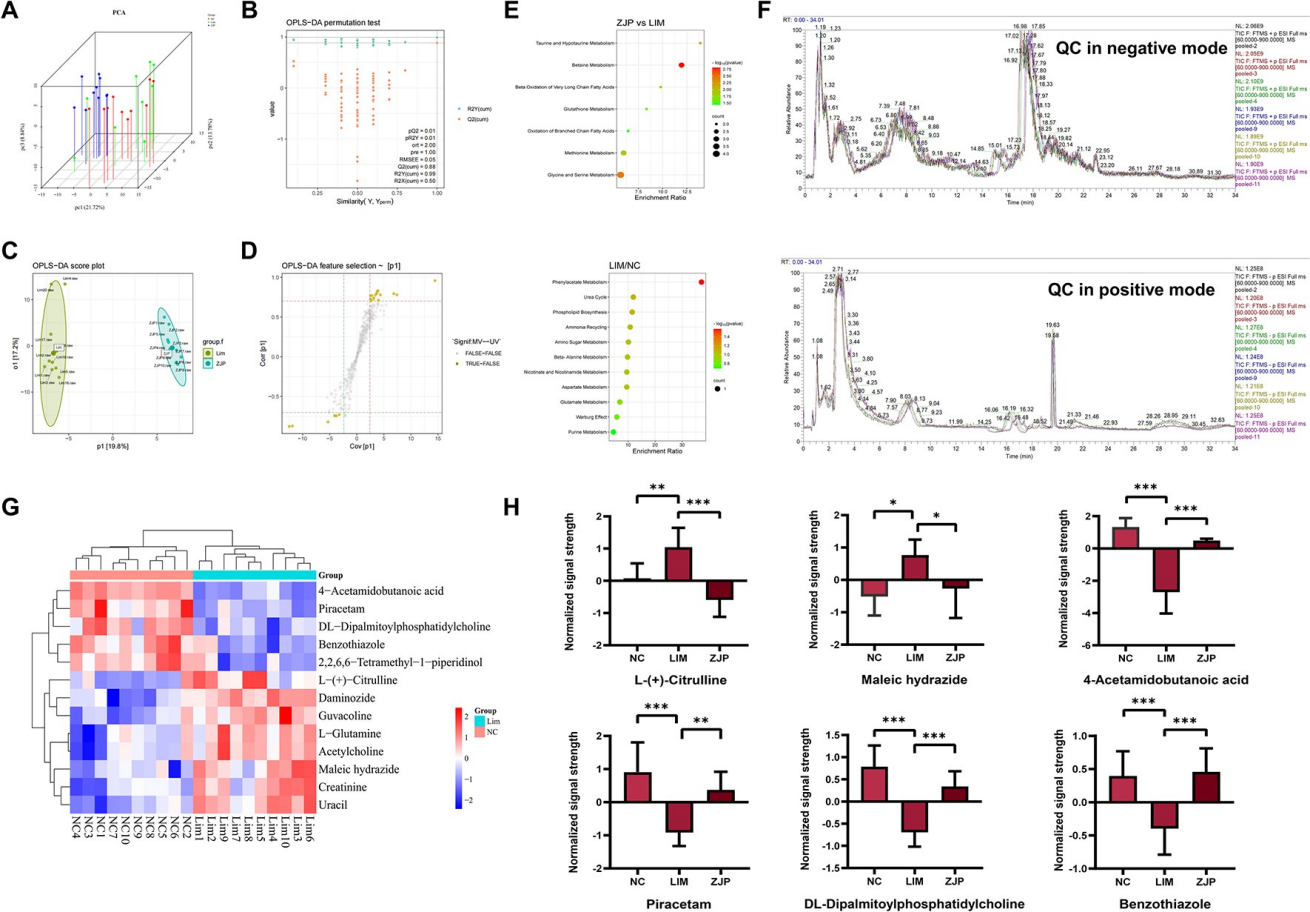
ZJP’s regulatory impact on the metabolic abnormality brought on by LIM. A, B: Validation plot and PCA score of serum samples obtained from various groups. C, D: Serum sample OPLS-DA score and S-plot from the ZJP and LIM groups. E: KEGG pathway analysis of differential metabolites. F: The total ion chromatogram of QC samples. G: The Cluster-heat map of differential metabolites selected by S-plot using Pearson distance. H: Comparison of the normalized signal strength of potential biomarkers.

To differentiate the metabolic profiles between the groups, PCA analysis was employed (Fig 2A). The NC, LIM, and ZJP samples among them all displayed separation. The permutation test, which showed that Q2 and R2 values were less than the initial points (Q2 = 0.88, R2Y = 0.99, and R2X = 0.50) (Fig 2B), supported the created OPLS-DA model as successful. Potential biomarkers linked to the development of myopia were also screened using OPLS-DA (Fig 2C and 2D). The total ion chromatogram of QC samples is displayed in Fig 2F.

Candidate metabolites were screened using an S-plot (Fig 2D) with VIP > 1.0 and q < 0.05. The 13 metabolites selected using S-plot were shown as Cluster-heatmap using pearson distance (Fig 2G). Among the 13 metabolites, 5 were down-regulated in the LIM group, such as DL-dipalmitoylphosphatidylcholine, 4-acetamidobutanoic acid, and piracetam, while 8 were up-regulated, such as maleic hydrazide, L- (+)-citrulline, and cathine. In comparison to the LIM group, the level of the ZJP group up-regulated for DL-dipalmitoylphosphatidylcholine, 4-acetylaminobutyric acid, piracetam, and benzothiazole, down-regulated for the expression of L-(+)-citrulline and maleic hydrazide, reversing the expression trend of these five metabolites (Fig 2H).

**Fig 3:**
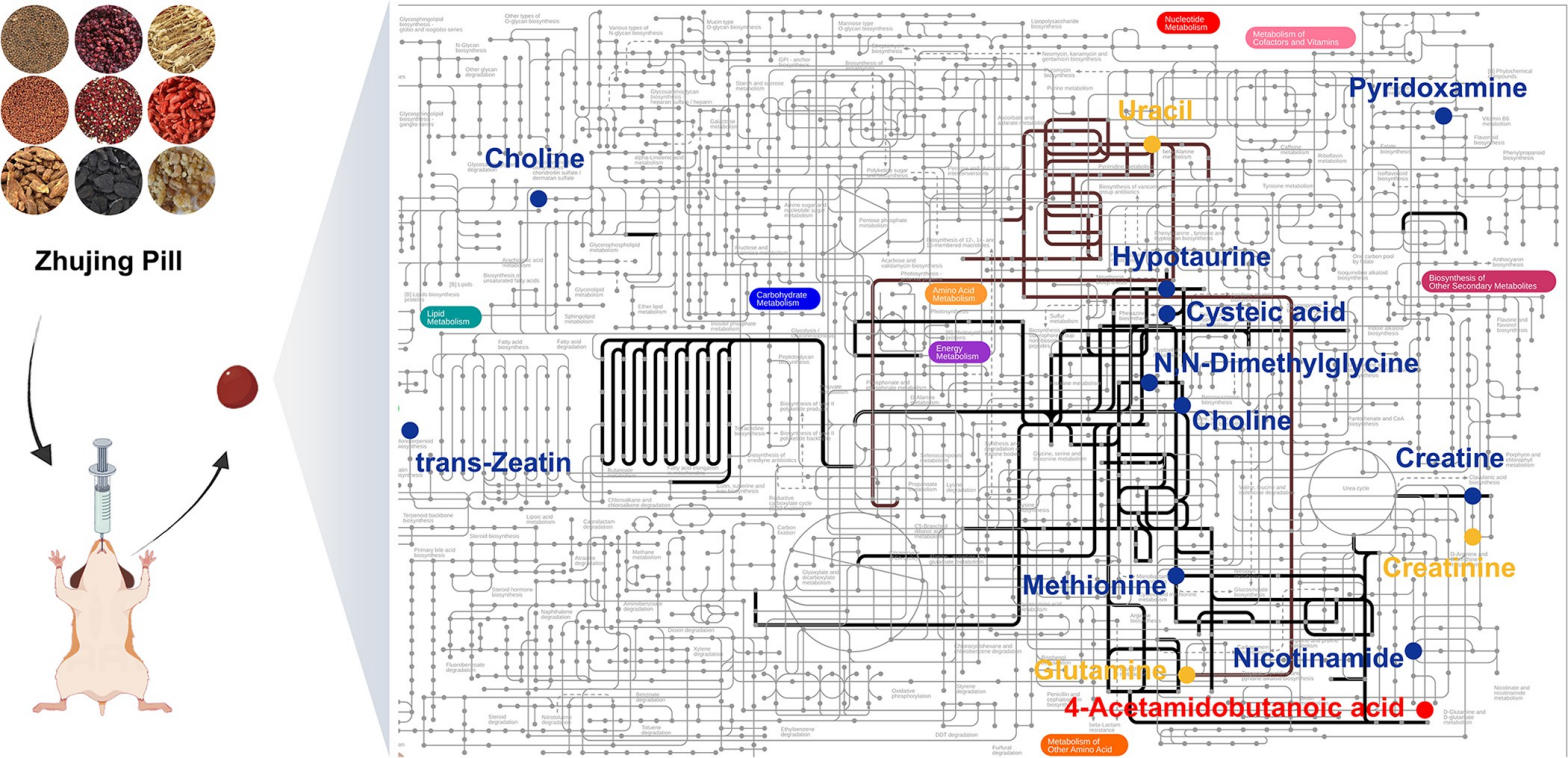
The potential mechanism through which ZJP suppresses myopia progression. The yellow and blue dots represent differential metabolites between the LIM and NC groups and between the ZJP and LIM groups, respectively. The red dot indicates the intersection of the two. The brown and black lines represent pathways of differential metabolite enrichment between the LIM and NC groups and between the ZJP and LIM groups, respectively.

For pathway enrichment analysis, we enter the metabolites that were expressed differently into MetaboAnalyst. According to KEGG pathway analysis, the LIM and NC groups exhibited significant differences in two pathways, including pyrimidine metabolism and phenylacetate metabolism (P<0.05) (Fig 2E). There are several pathways with significant differences between the ZJP and LIM groups that are enriched in betaine metabolism, methionine metabolism, glycine and serine metabolism, and phosphatidylethanolamine biosynthesis (P<0.05). In summary, metabonomics reveals the potential mechanism by which ZJP slows down the progression of myopia (Fig 3).

### Network pharmacology

**Fig 4:**
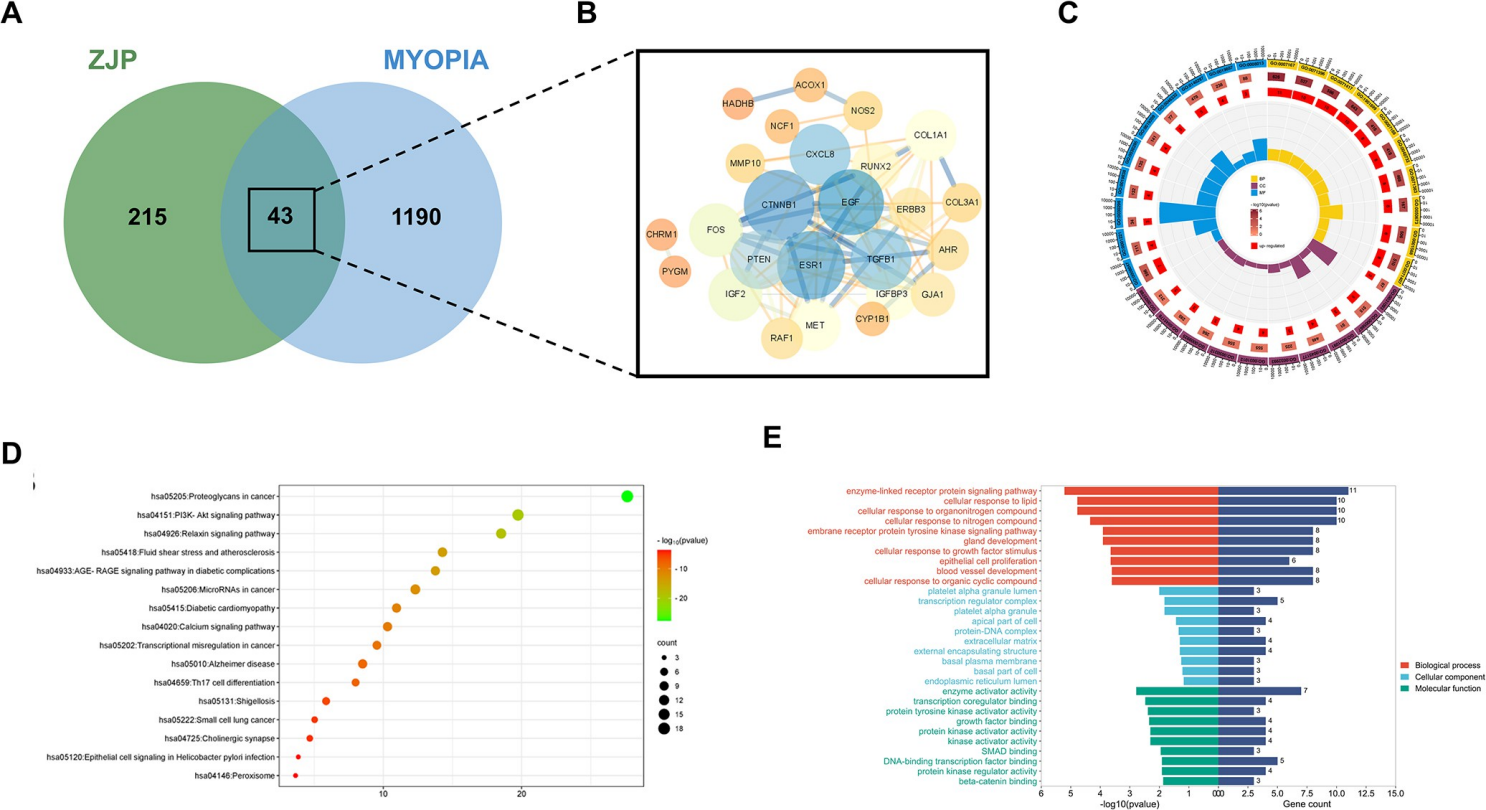
Venn diagram analysis and KEGG pathway annotation. A: Venn diagrams of the ZJP target and myopia target. B: The anti-myopic target interaction network of ZJP. C: GO enrichment analysis of differentially expressed genes. D: KEGG pathway analysis of ZJP anti-myopia targets obtained from network pharmacology. E: Summary of the results of enrichment analysis of anti-myopic targets by ZJP.

To investigate the mechanism of the inhibitory effect of ZJP on myopia progression, we employed network pharmacology. In our comprehensive investigation of ZJP, a total of 99 active components were identified based on stringent criteria of oral bioavailability (OB) ≥ 30% and drug-likeness (DL) ≥ 0.18. From these components, 258 potential ZJP targets were delineated through a process of high-probability screening and redundancy elimination. Employing the GeneCards database with a relevance score threshold of >1 and the Online Mendelian Inheritance in Man (OMIM) database, a search using "myopia" as the keyword yielded 1233 potential myopic targets. Subsequently, a cross-analysis between the list of ZJP targets and myopic targets resulted in the identification of 43 significant anti-myopic targets of ZJP, as illustrated in Fig 4A. Table 2 presents the interaction between 18 active ZJP components and these anti-myopic targets.

**Table 2:** Active ZJP components interacted with ZJP anti-myopic targets.

MOL ID	compound name	Protein name	Gene name	count
MOL000098	quercetin			30
		Stromelysin-1	MMP3	
		Epidermal growth factor receptor	EGFR	
		RAC-alpha serine/threonine-protein kinase	AKT1	
		Vascular endothelial growth factor A	VEGFA	
		Proto-oncogene c-Fos	FOS	
		72 kDa type IV collagenase	MMP2	
		Matrix metalloproteinase-9	MMP9	
		Interleukin-10	IL10	
		Pro-epidermal growth factor	EGF	
		Tumor necrosis factor	TNF	
		Interleukin-6	IL6	
		Cellular tumor antigen p53	TP53	
		RAF proto-oncogene serine/threonine-protein kinase	RAF1	
		Superoxide dismutase [Cu-Zn]	SOD1	
		Interstitial collagenase	MMP1	
		Hypoxia-inducible factor 1-alpha	HIF1A	
		Gap junction alpha-1 protein	GJA1	
		Interleukin-8	CXCL8	
		Transforming growth factor beta-1	TGFB1	
		Cytochrome P450 1B1	CYP1B1	
		Collagen alpha-1(I) chain	COL1A1	
		Arachidonate 5-lipoxygenase	PTEN	
		Phosphatidylinositol-3,4,5-trisphosphate 3-phosphatase and dual-specificity protein phosphatase PTEN	PTEN	
		Neutrophil cytosol factor 1	NCF1	
		Aryl hydrocarbon receptor	AHR	
		Collagen alpha-1(III) chain	COL3A1	
		Runt-related transcription factor 2	RUNX2	
		Insulin-like growth factor-binding protein 3	IGFBP3	
		Insulin-like growth factor II	IGF2	
		Receptor tyrosine-protein kinase erbB-3	ERBB3	
MOL000006	luteolin			13
		Epidermal growth factor receptor	EGFR	
		RAC-alpha serine/threonine-protein kinase	AKT1	
		Vascular endothelial growth factor A	VEGFA	
		72 kDa type IV collagenase	MMP2	
		Matrix metalloproteinase-9	MMP9	
		Interleukin-10	IL10	
		Tumor necrosis factor	TNF	
		Interleukin-6	IL6	
		Cellular tumor antigen p53	TP53	
		Interstitial collagenase	MMP1	
		Induced myeloid leukemia cell differentiation protein Mcl-1	MCL1	
		Tyrosinase	TYR	
		Hepatocyte growth factor receptor	MET	
MOL002773	beta-carotene			7
		RAC-alpha serine/threonine-protein kinase	AKT1	
		Vascular endothelial growth factor A	VEGFA	
		72 kDa type IV collagenase	MMP2	
		Interstitial collagenase	MMP1	
		Catenin beta-1	CTNNB1	
		Gap junction alpha-1 protein	GJA1	
		Stromelysin-2	MMP10	
MOL000422	kaempferol			7
		Nitric oxide synthase, inducible	NOS2	
		Muscarinic acetylcholine receptor M1	CHRM1	
		RAC-alpha serine/threonine-protein kinase	AKT1	
		Tumor necrosis factor	TNF	
		Interstitial collagenase	MMP1	
		Cytochrome P450 1B1	CYP1B1	
		Aryl hydrocarbon receptor	AHR	
MOL000546	diosgenin			6
		RAC-alpha serine/threonine-protein kinase	AKT1	
		Vascular endothelial growth factor A	VEGFA	
		Cellular tumor antigen p53	TP53	
		Superoxide dismutase [Cu-Zn]	SOD1	
		Hypoxia-inducible factor 1-alpha	HIF1A	
		Serine/threonine-protein kinase mTOR	MTOR	
MOL000354	isorhamnetin			4
		Nitric oxide synthase, inducible	NOS2	
		Estrogen receptor	ESR1	
		Glycogen phosphorylase, muscle form	PYGM	
		Neutrophil cytosol factor 1	NCF1	
MOL001558	sesamin			3
		Interleukin-10	IL10	
		Peroxisomal acyl-coenzyme A oxidase 1	ACOX1	
		Trifunctional enzyme subunit beta, mitochondrial	HADHB	
MOL005944	matrine			3
		72 kDa type IV collagenase	MMP2	
		Tumor necrosis factor	TNF	
		Interleukin-6	IL6	
MOL000787	Fumarine			3
		Muscarinic acetylcholine receptor M3	CHRM3	
		Muscarinic acetylcholine receptor M1	CHRM1	
		Vascular endothelial growth factor receptor 2	KDR	
MOL000358	beta-sitosterol			2
		Muscarinic acetylcholine receptor M3	CHRM3	
		Muscarinic acetylcholine receptor M1	CHRM1	
MOL005406	atropine			2
		Muscarinic acetylcholine receptor M3	CHRM3	
		Muscarinic acetylcholine receptor M1	CHRM1	
MOL008400	glycitein			2
		Estrogen receptor	ESR1	
		Nitric oxide synthase, inducible	NOS2	
MOL009650	Atropine			2
		Muscarinic acetylcholine receptor M3	CHRM3	
		Muscarinic acetylcholine receptor M1	CHRM1	
MOL001243	3alpha-Hydroxy-olean-12-en-24-oic-acid			2
		Muscarinic acetylcholine receptor M3	CHRM3	
		Muscarinic acetylcholine receptor M1	CHRM1	
MOL001297	trans-gondoic acid			1
		Muscarinic acetylcholine receptor M1	CHRM1	
MOL004624	Longikaurin A			1
		Muscarinic acetylcholine receptor M1	CHRM1	
MOL008978	Gomisin R			1
		Estrogen receptor	ESR1	
MOL001215	tirucallol			1
		Muscarinic acetylcholine receptor M3	CHRM3	

The constructed Protein-Protein Interaction (PPI) network pinpointed catenin beta 1 (CTNNB1), estrogen receptor 1 (ESR1), and epidermal growth factor (EGF) as central hub targets in ZJP’s mechanism against myopia, depicted in Fig 4B. Analysis of Gene Ontology (GO) categories revealed that ZJP anti-myopic targets predominantly participate in 291 biological processes (GO-BP), such as cellular response to lipids and enzyme-linked receptor protein signaling pathways. Moreover, within the molecular function (GO-MF) domain, these targets are chiefly involved in 22 functions, including enzyme activator activity and transcription coregulator binding. In the cellular component (GO-CC) category, significant enrichment annotations included the platelet alpha granule and transcription regulator complex, as shown in Fig 4C and 4E. Further elucidation through Kyoto Encyclopedia of Genes and Genomes (KEGG) pathway enrichment analysis indicated that ZJP potentially counteracts myopia primarily via pathways such as proteoglycans in cancer and the PI3K-Akt signaling pathway, among others. Fig 4D displays 16 pathways that were significantly enriched based on KEGG annotation, offering a deeper insight into the complex mechanisms by which ZJP might exert its therapeutic effects on myopia.

### Analysis of combined metabonomics and network pharmacology

**Fig 5:**
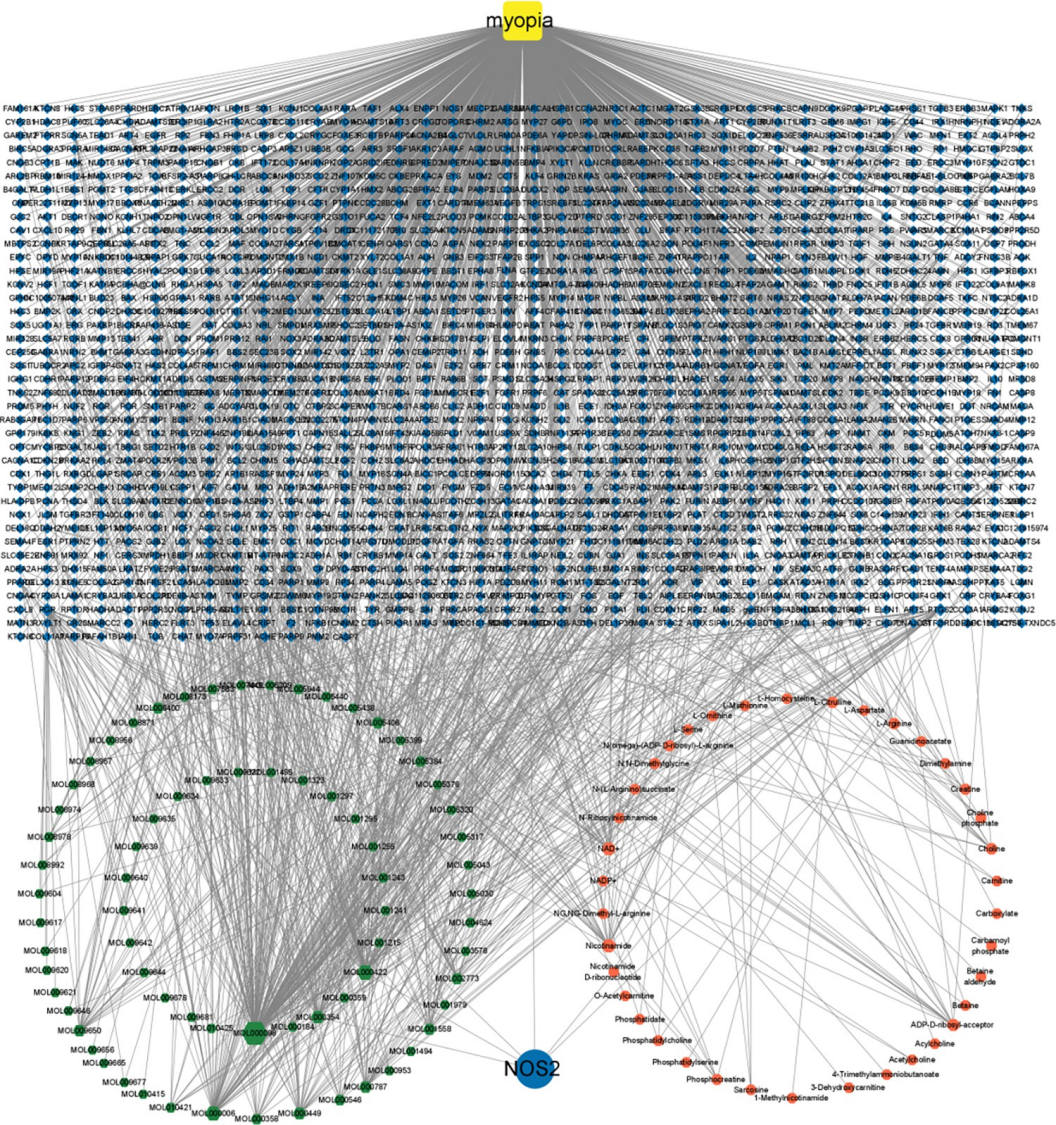
Construction of the compound-gene-disease-metabolites network. Green regular hexagons represent compounds, yellow squares represent disease, and the red octagon represents metabolites. The blue diamond represents genes, including targets corresponding to potential active components, disease-related targets, and targets corresponding to differential metabolites. The blue circle represents the intersection of network pharmacology and metabolomics genes.

To fully understand the anti-myopia role of ZJP, differential metabolites related to the anti-myopia of ZJP obtained from serum metabonomics were introduced into Metscape (plug-in in Cytoscape software). Finally, 57 target genes were obtained based on these seven differential metabolites, and the compound-gene-disease-metabolite network was constructed (Fig 5). We discovered that NOS2 is the core target by intersecting the anti-myopic targets of ZJP identified by network pharmacology with those identified by metabonomics.

LPCAT1 is the synthase of DL-Dipalmitoylphosphatidylcholine in the retina [9]. CHRNA7 can regulate the release of Gamma-Aminobutyric Acid (GABA) in the retina [10], which is related to the change in the level of 4-Acetamidobutanoic acid, the metabolic derivative of GABA. Therefore, we regard NOS2, LPCAT1, and CHRNA7 as the core targets of ZJP for anti-myopia. Table 3 demonstrates the active components of ZJP related to core and hub targets derived by PPI based on network pharmacology (Table 3).

**Table 3:** Active ZJP components interacted with core and hub targets.

Gene name	Protein name	MOLID	Compound name
NOS2	Nitric oxide synthase, inducible	MOL008400	glycitein
		MOL000354	isorhamnetin
		MOL000422	kaempferol
CHRNA7	Neuronal acetylcholine receptor protein, alpha-7 chain	MOL001297	trans-gondoic acid
		MOL000449	Stigmasterol
LPCAT1	Lysophosphatidylcholine acyltransferase1	/	/
ESR1	Estrogen receptor	MOL008400	glycitein
		MOL008978	Gomisin R
		MOL000354	isorhamnetin
EGF	Pro-epidermal growth factor	MOL000098	quercetin
CTNNB1	Catenin beta-1	MOL002773	beta-carotene

### NOS2, LPCAT1, and CHRNA7 expression

**Fig 6:**
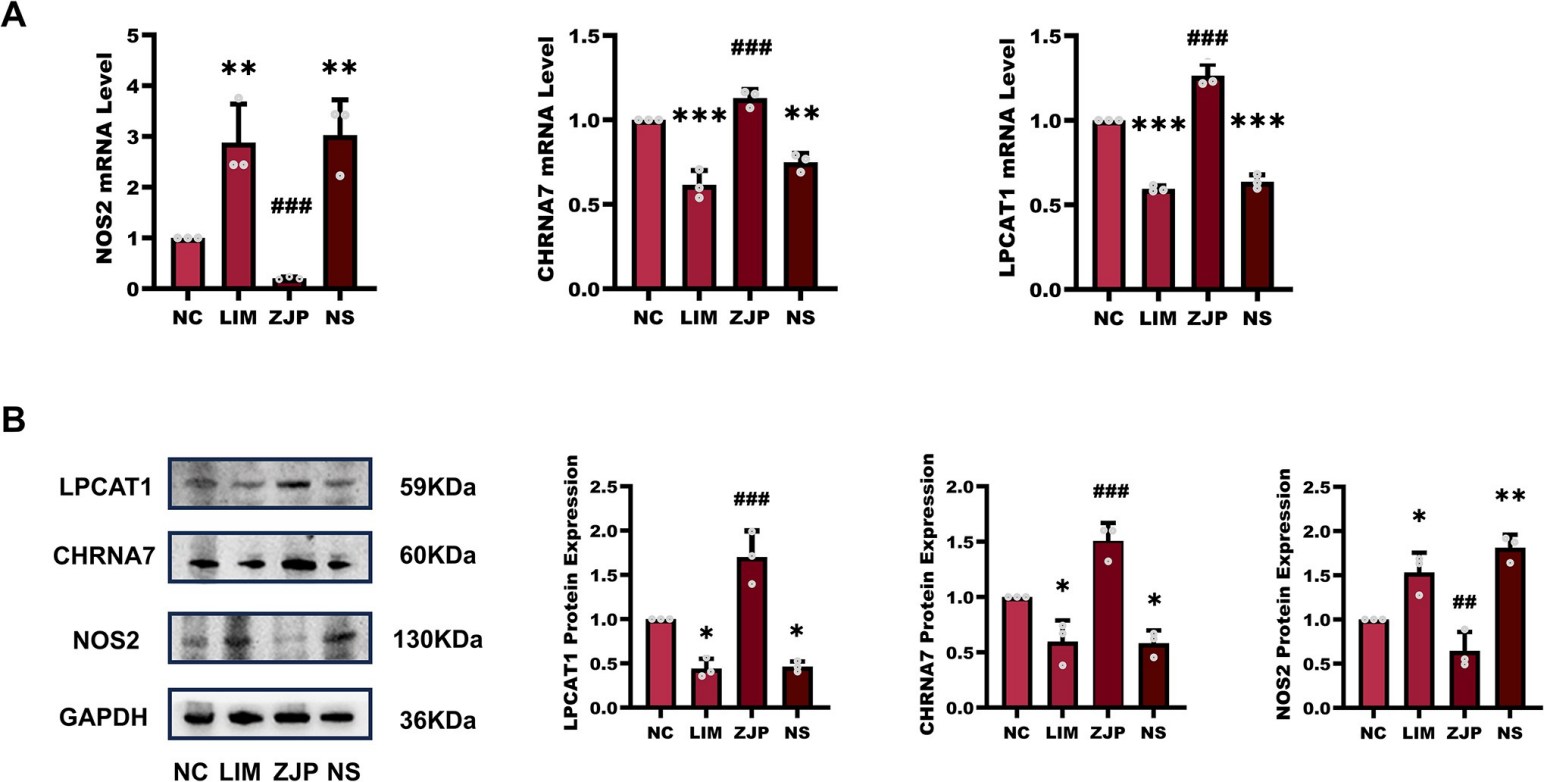
The expression of NOS2, LPCAT1, and CHRNA7. A: The mRNA levels of the NOS2, LPCAT1, and CHRNA7 genes were identified by qPCR in the retinal tissues in the NC, LIM, ZJP, and NS groups. B: Western blot analysis of LPCAT1, CHRNA7, and NOS2. *P < 0.05, **P < 0.01 and ***P < 0.001 vs. the NC group, ^##^P < 0.05 and ^###^P < 0.001 vs. the LIM group.

To verify core targets of ZJP for anti-myopia, we analyzed their expression by qPCR and western blot. Fig 6A illustrates that following myopia induction for four weeks, the LIM group’s NOS2 gene expression rose in comparison to the NC group, and this difference was statistically significant (P<0.01). Following myopia induction for 4 weeks (P<0.001), NOS2 gene expression was lower in the ZJP group compared to the LIM group. In contrast, the LPCAT1 gene showed a significant drop in expression following a 4-week induction of myopia (P<0.001). The ZJP group’s LPCAT1 gene expression increased in comparison to the LIM group (P<0.001). Similarly, following 4 weeks of myopia induction, CHRNA7 gene expression fell significantly (P<0.001). However, in the ZJP group, CHRNA7 gene expression increased compared to the LIM group. (P<0.001).

Fig 6B demonstrated that, following myopia induction for four weeks, NOS2 protein expression was up-regulated in comparison to the NC group (P<0.05), whereas NOS2 expression was down-regulated in the ZJP groups (P<0.01). By contrast, compared with the NC group, the levels of LPCAT1 (P<0.05) and CHRNA7 (P<0.05) protein decreased after myopia induction for 4 weeks. The protein levels of LPCAT1 (P<0.001) and CHRNA7 (P<0.001) in the ZJP group were higher than the levels of the LIM group.

## Discussion

The enzyme inducible nitric oxide synthase (iNOS) is known to catalyze the transformation of arginine into citrulline and nitric oxide (NO), a process detailed by Mori and Gotoh (2004) [11]. Metabonomic analyses revealed that serum levels of L-(+)-citrulline were elevated in the LIM group compared to the NC group, but notably decreased following treatment with ZJP. This observation aligns with metabonomic findings from a study on human subjects with high myopia and cataracts, which indicated increased levels of arginine and citrulline in the aqueous humor of those with high myopia [12]. Consistent with these metabonomic results, we observed an upregulation in the expression of iNOS mRNA and protein in the LIM group, whereas such expressions were mitigated in the ZJP-treated group.

Supporting the link between nitric oxide synthase activity and myopia progression, intravitreal administration of N-nitro-L-arginine methyl ester (L-NAME), a nitric oxide synthase inhibitor, has been shown to decelerate the progression of myopia in LIM chicks [13] and to increase NOS activity along with cyclic guanosine monophosphate (cGMP) levels in chronic form-deprivation myopia (FDM) guinea pigs [14]. Consequently, our findings demonstrate that in the LIM group, there is an upregulation of iNOS expression and a concomitant increase in citrulline level, suggesting that NO contributes to the development of myopia. Conversely, ZJP treatment appears to counteract the progression of myopia by modulating iNOS expression and reducing NO levels, thereby highlighting a potential therapeutic mechanism of ZJP in myopia management.

4-Acetylaminobutyric acid, an acetylated derivative of GABA, exhibits potential antioxidative properties [15]. Metabonomic analysis indicated a decrease in the serum levels of 4-acetylaminobutyric acid in the LIM group compared to the NC group, with levels subsequently rising upon treatment with ZJP. This contrasts with observations that GABA levels significantly increase in the retina of LIM models, suggesting that ZJP may counteract myopia development by modulating GABA acetylation.

The GABA receptor agonist, Baclofen, has been documented to reduce LIM [16]. CHRNA7, a subtype of the nicotinic acetylcholine receptor composed entirely of 7 subunits, is known to facilitate the release of GABA vesicles in the retina. Activation of CHRNA7 enhances the activity of GABAA receptors in retinal ganglion cells and GABAB receptors in retinal capillary endothelial cells [10]. In the LIM model, CHRNA7 mRNA and protein expression levels were found to decrease, yet these levels were elevated in the ZJP-treated group. The diminished expression of CHRNA7 in the LIM model did not lead to a reduction in retinal GABA levels [17], indicating that the decreasing CHRNA7 expression could not directly contribute to myopia progression by reducing GABA levels, but might involve the regulation of GABA receptor activity among other mechanisms.

Nicotinic receptor agonists have been shown to prevent myopia development, suggesting that CHRNA7 activation may impede myopia progression [18]. Given that retinal dopaminergic neurons concurrently release dopamine and GABA, an imbalance in these neurotransmitters’ release could occur in myopia [19]. While dopamine levels decrease in the LIM model, CHRNA7 activation could potentially inhibit myopia by enhancing retinal dopamine content. Nonetheless, nicotine did not influence the reduction in dopamine and 3,4-dihydroxyphenylacetic acid (DOPAC) levels in form deprivation myopia (FDM) [18], suggesting that changes in dopamine content in FDM might not explain why decreased CHRNA7 expression fosters myopia development.

Dipalmitoyl Phosphatidylcholine (DPPC) is a major component of pulmonary surfactant and plays a critical role in the formation of retinol ester in the retina [20–22]. Metabonomic analysis revealed that, relative to the NC group, the DL-DPPC content in the serum of the LIM group decreased, whereas it increased following ZJP treatment. This finding aligns with research showing that glycerol phospholipid metabolic pathways, including phosphatidylcholine families, are associated with myopia in children and adolescents [23].

LPCAT1 (also known as AYTL2), a key enzyme in phospholipid biosynthesis/remodeling, facilitates the conversion of palmitoyllysophosphatidylcholine to DPPC, crucial for retinal photoreceptor homeostasis. Its deficiency in rd11 mice leads to photoreceptor degradation [9]. Our data reveal a decrease in LPCAT1 mRNA and protein expression in the LIM group, with an increase observed upon ZJP treatment, underscoring the potential mechanisms through which ZJP might exert protective effects against myopia.

Light-induced activation of retinal dopaminergic amacrine cells plays a pivotal role in visual signaling processes, as demonstrated in several studies [24,25]. Additionally, mice harboring mutations that lead to photoreceptor degeneration exhibit diminished levels of dopamine (DA) and its metabolite DOPAC throughout normal development, making them more susceptible to form FDM [26]. These observations suggest that a disruption in photoreceptor homeostasis, potentially driven by decreased LPCAT1 expression, could exacerbate myopia development by reducing DA levels.

CTNNB1, a key component in the growth and survival of retinal pigment epithelial (RPE) cells, functions not only as a structural element of intercellular junctions but also as a transcriptional regulator within the Wnt signaling pathway, highlighting its significance in epithelial cell integrity, including that of RPE cells [27]. Investigations have shown that reduced serum levels of niclosamide (DKK-1), a Wnt pathway inhibitor, correlate with myopia, and that activation of the canonical Wnt pathway is a feature of myopia progression in mice. Inhibiting the canonical Wnt signaling pathway through DKK1 leads to reduced axial length elongation, thereby attenuating myopia progression [28]. Thus, CTNNB1’s role in myopia may be attributed to its influence on the Wnt signaling pathway.

Network pharmacology analyses have revealed that ZJP targets associated with anti-myopic effects are significantly enriched in pathways related to cancer, such as proteoglycans in cancer, microRNAs in cancer, transcriptional misregulation in cancer, and small cell lung cancer. ESR1, encoding estrogen receptor alpha (ERα), has been identified as a central protein in various diseases, including myeloid leukemia, where it serves as a pivotal communication hub [29]. Furthermore, the ERα-HIF-1α interaction in prostate cancer cells prevents the ubiquitination and subsequent degradation of HIF-1α, highlighting HIF-1α’s significance in scleral remodeling during myopia development [30]. Additionally, Cytochrome P450 1B1 influences ocular redox homeostasis and oxidative stress by modulating the ERα/BMP6/ferrimodulin axis in retinal endothelial cells [31]. The central role of ESR1 in interacting with numerous ZJP anti-myopic targets underscores its potential importance in the mechanism by which ZJP mediates its anti-myopic effects.

Epidermal Growth Factor (EGF) is another critical molecule, part of the growth factor ligands and receptors family, known for its involvement in wound healing and cell proliferation by binding to the epidermal growth factor receptor (EGFR) [32]. Research indicating that intravitreal injections of EGF and EGFR antibodies significantly reduced the axial length in LIM guinea pigs, with the variance in axial length between eyes decreasing as doses of the antibodies increased, suggests that ZJP’s myopia-inhibitory effects might also involve suppression of EGF expression [33]. These mechanisms collectively provide insight into the multifaceted approach ZJP employs to combat myopia progression.

Although ZLP was administered orally, there may be no changes of the NOS2, CHRNA7, and LPCAT1 in the retinal tissue of both eyes of the ZLP group. We have found that Longdan Xiegan Decoction, a traditional Chinese formulation for the treatment of autoimmune uveitis, could exert a specifically inhibitory effect on antigen-specific autoreactive T cells from EAU rats rather than CD4-induced Th17 response in healthy rats [34], indicating that there is no significant difference of disease-related genes in normal tissue after medication intervention. As in our previous studies [35,36], we also observed no significant difference in the axial length of the left eyes of guinea pigs between NC and LIM groups, and they were all in a state of non-myopia. Thus, based on these findings, we did not investigate these molecules in the present study.

## Conclusion

This research harnessed the synergistic methodologies of network pharmacology and metabonomics to elucidate the intricate mechanisms by which ZJP mitigates myopia, focusing on identifying potential targets and active compounds, alongside assessing changes in serum metabolites in myopic guinea pigs. Through the integrative analysis of network pharmacology and metabonomics data, it was established that ZJP combats myopia through the interplay of various components, targets, and pathways. Significantly, this study unveiled novel insights into the changes in retinal CHRNA7 and LPCAT1 expression associated with myopia, which ZJP administration effectively reversed. In sum, the outcomes of this study advance our comprehension of the complex pathogenesis of myopia and highlight the potential of employing metabonomics and network pharmacology to unravel the underlying mechanisms of traditional Chinese medicine in treating this condition.

## Supporting information

S1 Raw data (ZIP)

S1 Raw images (PDF)
