# Supplementary material for: Inhibitory effect of Zhujing Pill on myopia progression: Mechanistic insights based on metabonomics and network pharmacology
Source: PLoS One. 2024 Dec 3;19(12):e0312379. doi: 10.1371/journal.pone.0312379 (PMC11614212; doi:10.1371/journal.pone.0312379)

Fig 6B LPCAT1-1

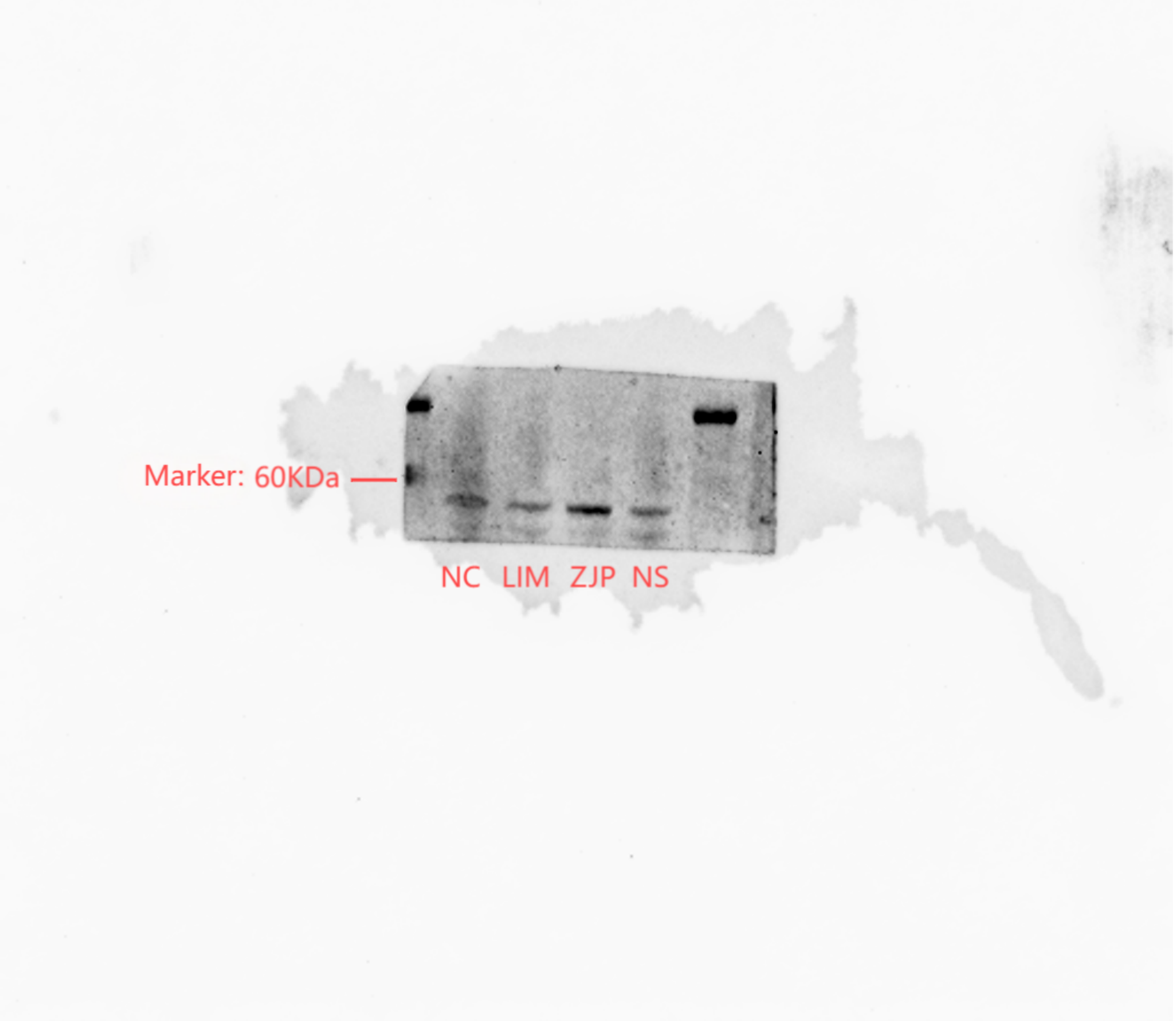

Fig 6B LPCAT1-2

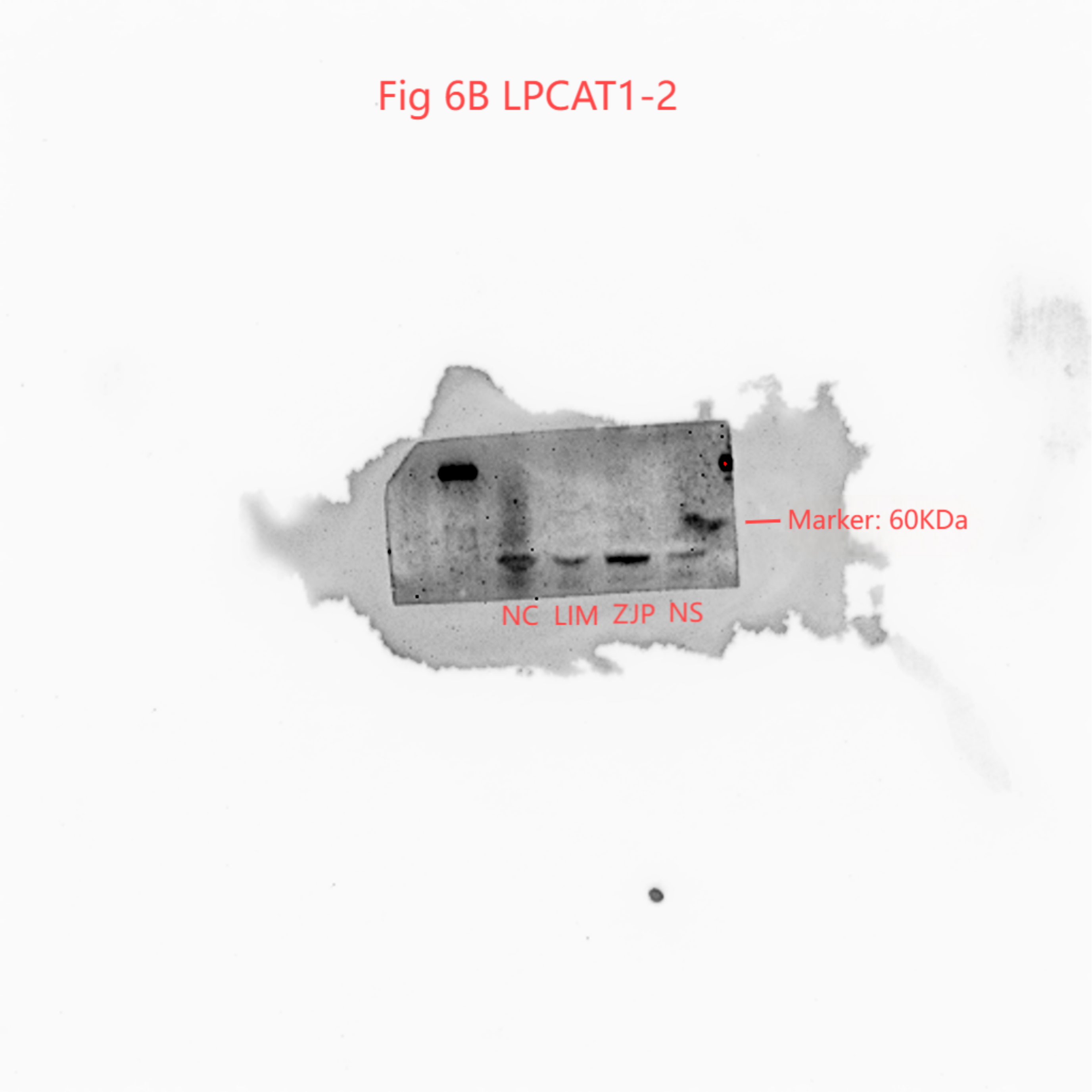

Fig 6B LPCAT1-3

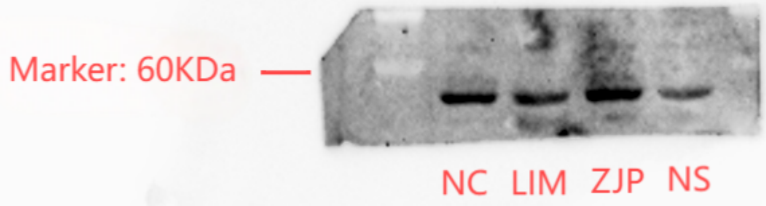

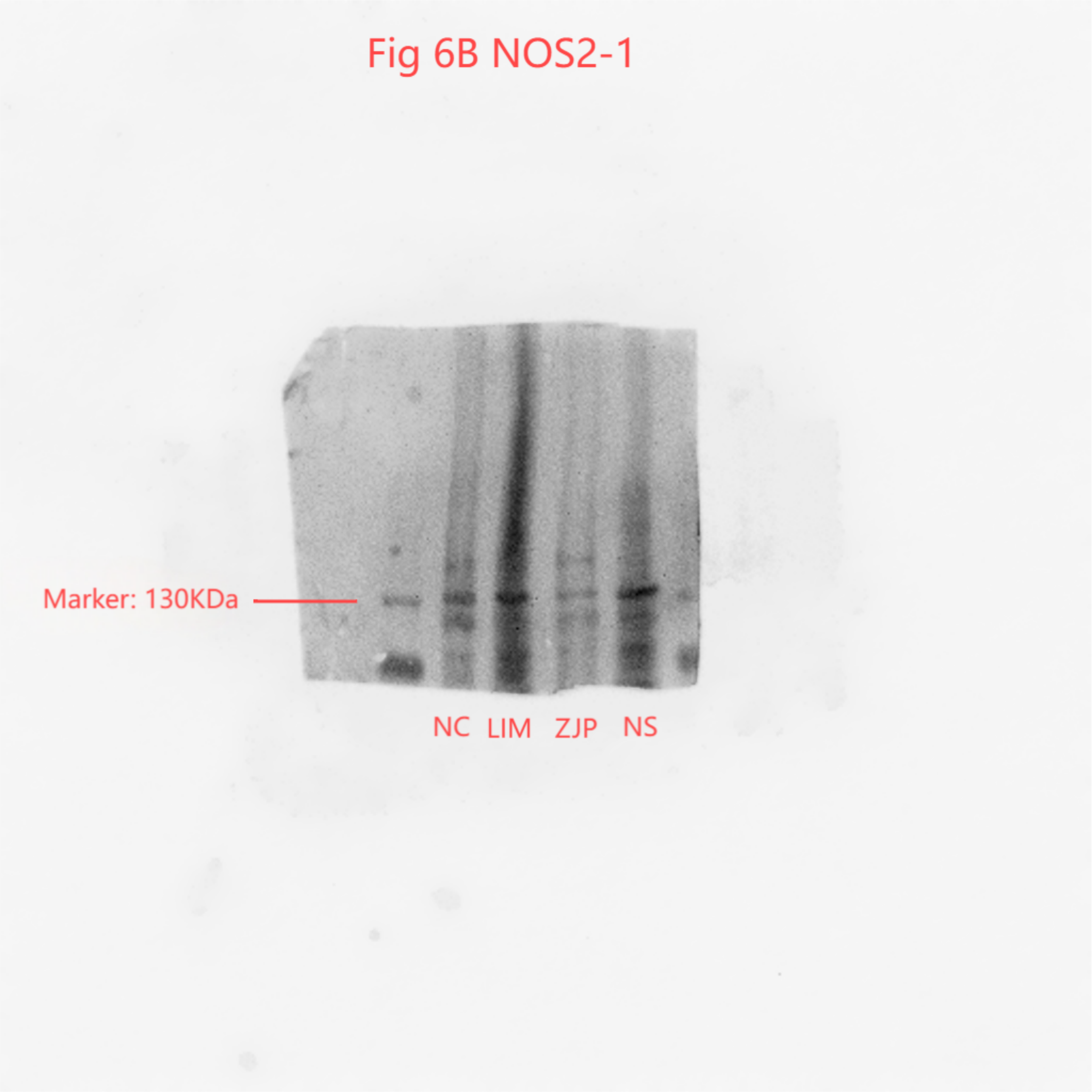
Fig 6B NOS2-1
Marker: 130KDa
NC
LIM
ZJP
NS

Fig 6B NOS2-2

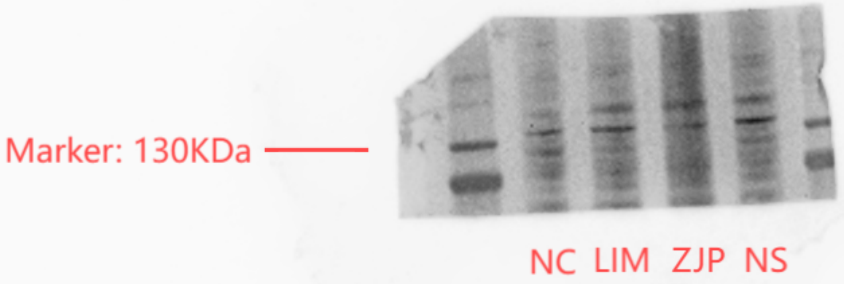

Fig 6B NOS2-3

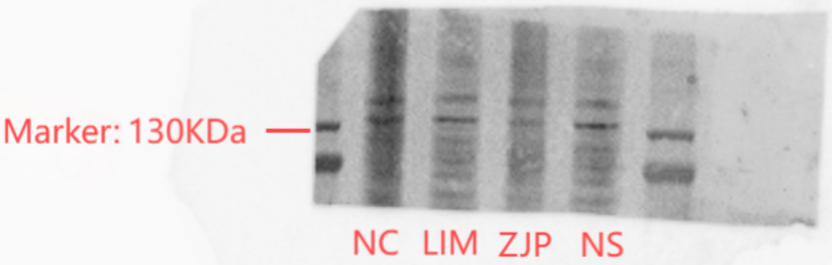

Fig 6B CHRNA7-1

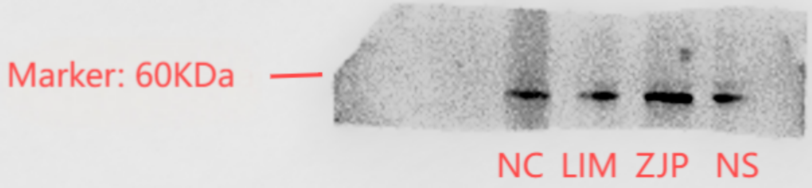

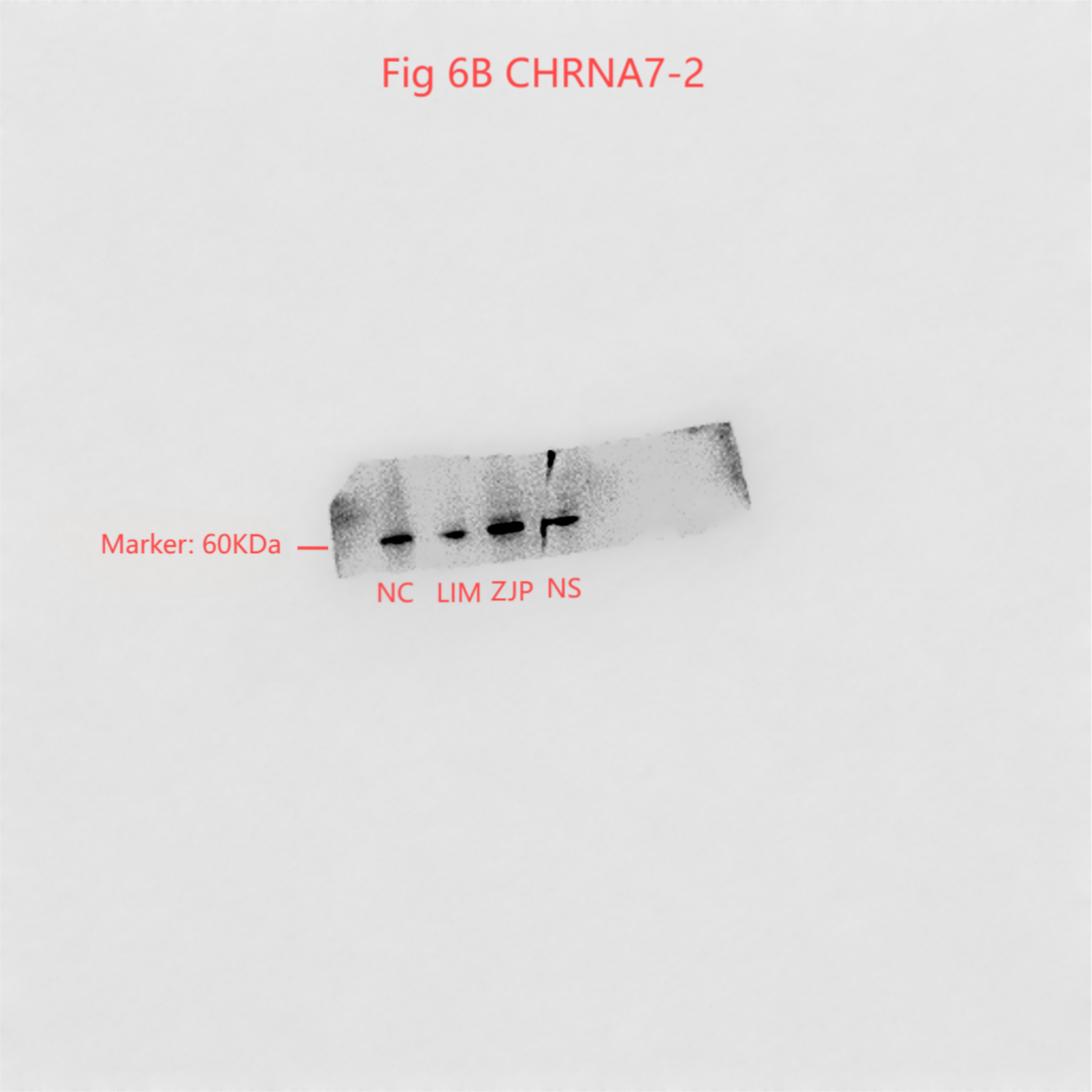
Fig 6B CHRNA7-2
Marker: 60KDa
NC
LIM
ZJP
NS

Fig 6B CHRNA7-3

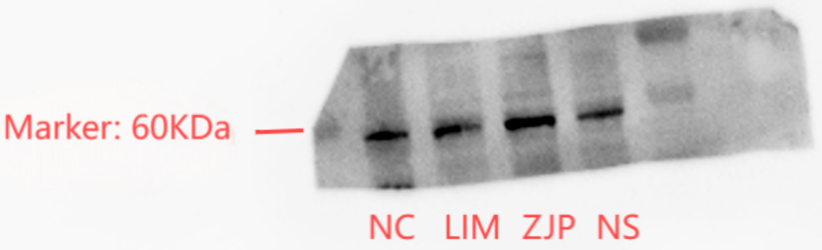

Fig 6B GAPDH-1

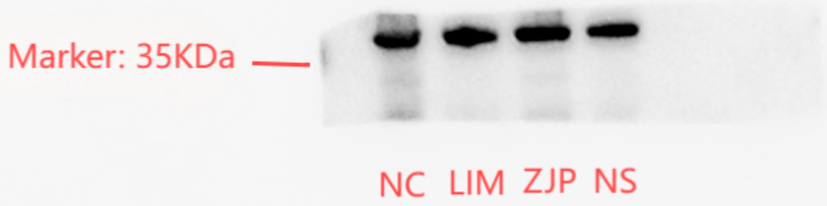

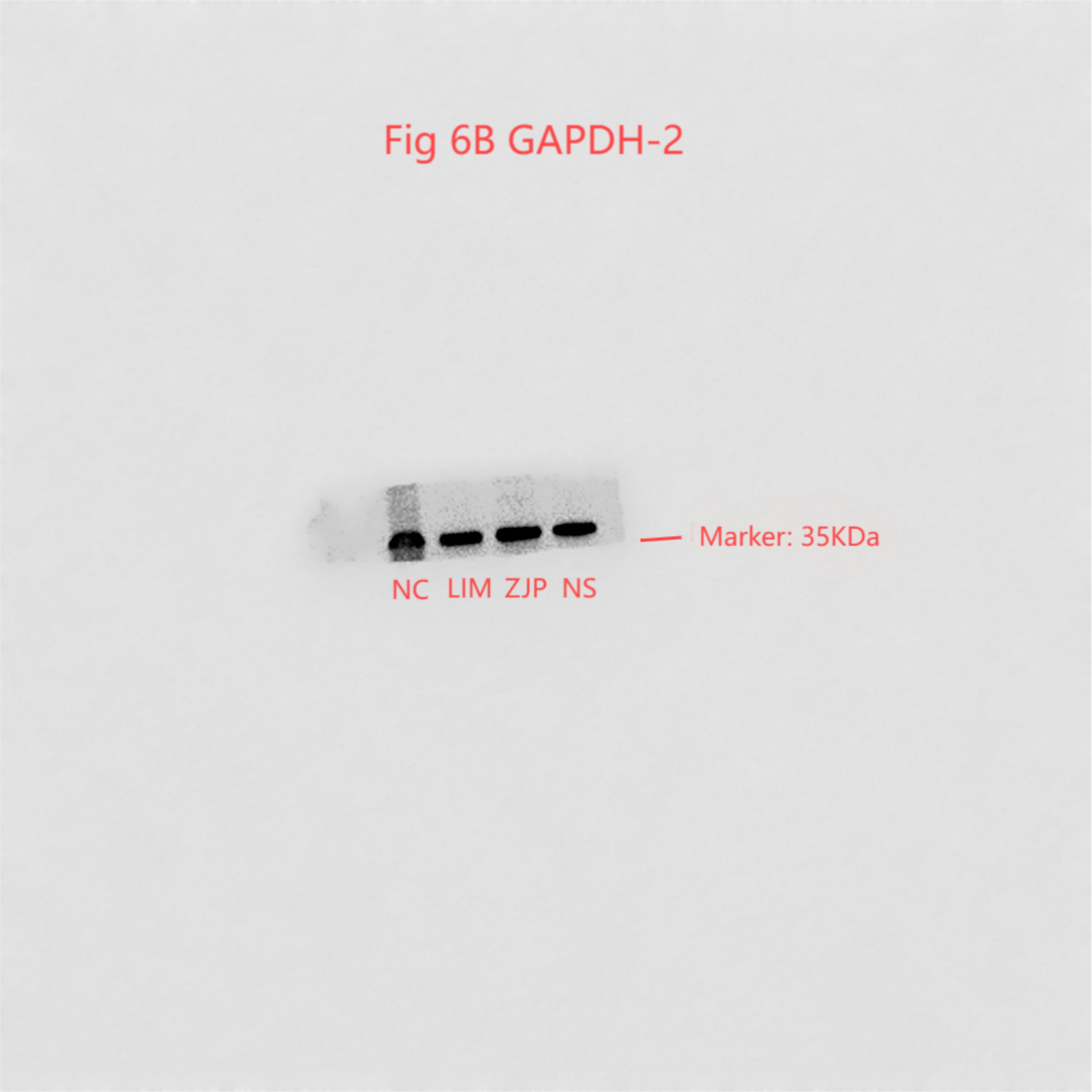
Fig 6B GAPDH-2
Marker: 35KDa
NC
LIM
ZJP
NS

Fig 6B GAPDH-3

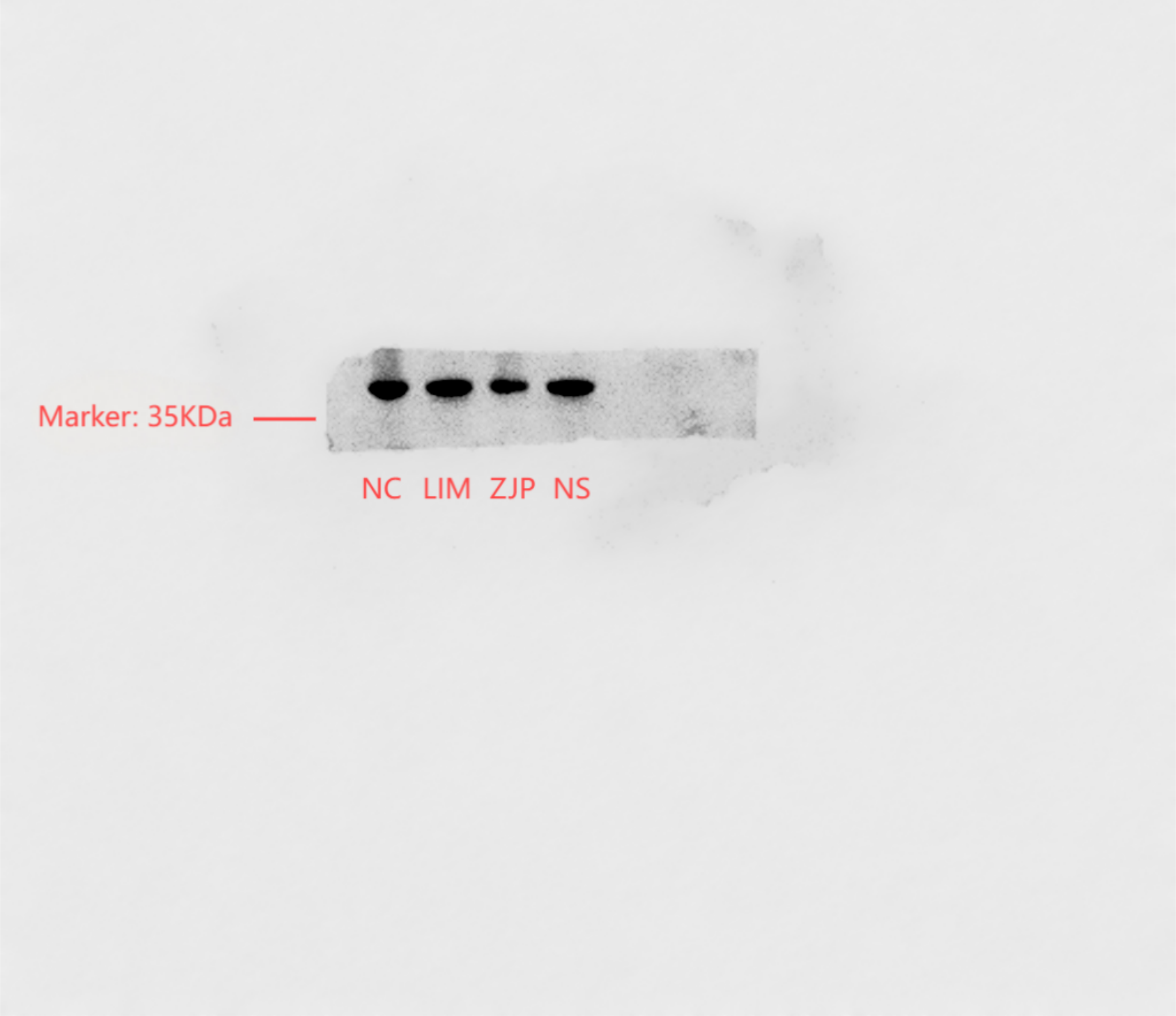

Supplement: S1 Raw images — (PDF) [file pone.0312379.s002.pdf]
